# PEG Reinforced Scaffold Promotes Uniform Distribution of Human MSC-Created Cartilage Matrix

**DOI:** 10.3390/gels8120794

**Published:** 2022-12-03

**Authors:** Kanyakorn Riewruja, Alyssa M. Aguglia, Sophie Hines, Meagan J. Makarcyzk, Sittisak Honsawek, Hang Lin

**Affiliations:** 1 Department of Orthopaedic Surgery, University of Pittsburgh School of Medicine, 450 Technology Drive, Rm 217, Pittsburgh, PA 15219, USA; 2 Osteoarthritis and Musculoskeleton, Faculty of Medicine, Chulalongkorn University, King Chulalongkorn Memorial Hospital, Thai Red Cross Society, Bangkok 10330, Thailand; 3 Department of Bioengineering, University of Pittsburgh Swanson School of Engineering, 450 Technology Drive, Rm 217, Pittsburgh, PA 15219, USA

**Keywords:** cartilage tissue engineering, hydrogel, mesenchymal stromal cells, migration

## Abstract

Previously, we used a gelatin/hyaluronic acid (GH)-based scaffold to induce chondrogenic differentiation of human bone marrow-derived mesenchymal stromal cells (hBMSC). The results showed that hBMSCs underwent robust chondrogenesis and facilitated *in vivo* cartilage regeneration. However, it was noticed that the GH scaffolds display a compressive modulus that is markedly lower than native cartilage. In this study, we aimed to enhance the mechanical strength of GH scaffolds without significantly impairing their chondrosupportive property. Specifically, polyethylene glycol diacrylate (PEGDA) and photoinitiators were infiltrated into pre-formed hBMSC-laden GH scaffolds and then photo-crosslinked. Results showed that infiltration of PEG at the beginning of chondrogenesis significantly increased the deposition of glycosaminoglycans (GAGs) in the central area of the scaffold. To explore the mechanism, we compared the cell migration and proliferation in the margin and central areas of GH and PEG-infiltrated GH scaffolds (GH+PEG). Limited cell migration was noticed in both groups, but more proliferating cells were observed in GH than in GH+PEG. Lastly, the in vitro repairing study with bovine cartilage explants showed that PEG- impregnated scaffolds integrated well with host tissues. These results indicate that PEG-GH hybrid scaffolds, created through infiltrating PEG into pre-formed GH scaffolds, display good integration capacity and represent a new tool for the repair of chondral injury.

## 1. Introduction

Articular cartilage is a structure within joints that provides a smooth, load-bearing surface for articulating bones. Once damaged, articular cartilage has little to no capability of self-repair. This causes pain and disability as well as an increased risk of developing early onset of osteoarthritis, which is problematic because younger patients are generally poor candidates for total knee arthroplasty [1].

Even though current strategies for cartilage repair have been developed over time, cartilage regeneration outcomes remain unpredictable. Microfracture was the long-established method to restore osteochondral defects, which is limited by fibrocartilage formation post treatment. As a result, repair methods that deliver cells into the defect directly while adhering to the proper environment are necessary. For example, autologous chondrocyte implantation (ACI) demonstrates favorable outcomes and better neo-tissue quality in comparison to microfracture [2,3,4], which however is limited by cell availability and injury to the donor sites.

Because of their chondrogenic potential, mesenchymal stem cells (MSC) have been considered as an alternative cell source to native chondrocytes for cell-based cartilage repair. Compared to chondrocytes, MSCs are easier to isolate, have greater proliferation potential, and provide more advantages in terms of trophic effects for chondrocyte regeneration [5]. Until now, numerous studies reported promising outcomes using MSCs in cartilage tissue engineering [6].

In addition to cells, biomaterial scaffolds are often needed to fill the avoid space of cartilage injury and also provide a microenvironment for chondrogenic differentiation. To date, no specific scaffold has achieved the ideal features that are identical to native hyaline cartilage. Synthetic scaffolds, such as poly (ethylene glycol) (PEG), provide high mechanical strength, yet they do not contain cell-binding motifs; thus, cell proliferation and tissue integration are limited [7]. Alternatively, natural scaffolds, such as collagen, gelatin, or hyaluronic acid, better promote chondrogenic conduction but usually display low mechanical strength. For example, gelatin is a denatured form of collagen that has been previously studied in our lab to support chondrogenic differentiation of human bone marrow-derived MSCs (hBMSCs) [8]. Its chondrosupportive function was further improved by incorporating hyaluronic acid (HA), which is an important component in the native cartilage ECM [9]. In this study, the cartilage repairing capacity of gelatin and HA combined scaffold (GH) was also examined in rabbits.

In order to develop a new scaffold with appropriate mechanical properties to native articular cartilage and the ability to adequately support chondrogenesis, we combined two PEG and GH-based scaffolds. Instead of directly mixing them before polymerization, we adapted the method that we recently used [10], in which uncured monomer and photoinitiators were infiltrated into pre-formed GH hydrogel and then in situ photopolymerized within cartilage. The new method allowed for the incorporation of uncured poly (ethylene glycol) diacrylate (PEGDA) at the beginning and also at any time during chondrogenesis. Using this novel technology, we introduced PEGDA at day 0, 7, 14, or 21 of chondrogenic culture to examine the influences on hBMSCs chondrogenesis within GH scaffolds. Next, attempting to explore the mechanism of how PEG incorporation affects hBMSCs chondrogenesis, we assessed the cell migration and proliferation within different scaffolds. Lastly, an in vitro repair study was conducted to examine the capacity of GH+PEG hybrid scaffolds in regenerating new cartilage.

## 2. Results

### 2.1. PEG Hydrogel Is Able to Polymerize within the Pre-Formed Gelatin-Hyaluronic Acid (GH) Scaffold

**Figure 1:**
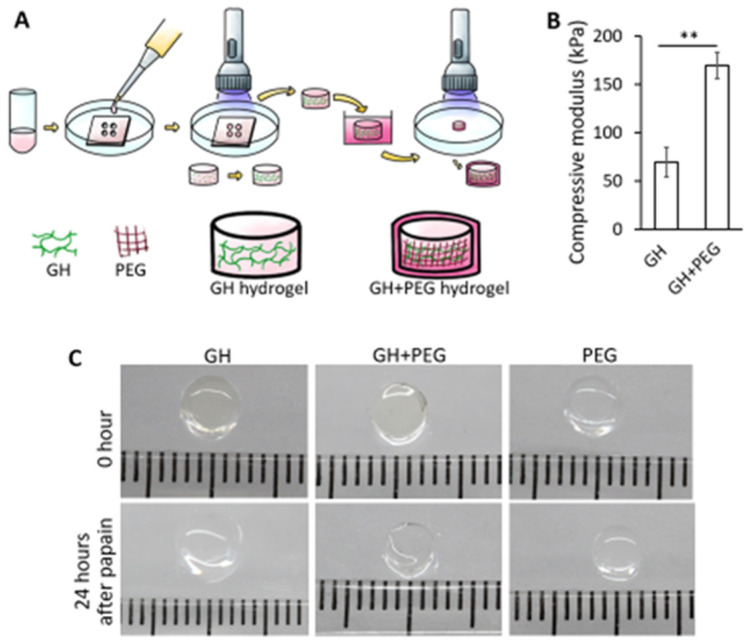
(**A**). The process of generating PEG-infiltrated GH (GH+PEG) scaffold. Specifically, uncured gelatin and hyaluronic acid (GH)/LAP (photoinitiator) solution were added into a mold with a cylindrical void space, then subjected to visible light illumination. Next, the pre-formed GH scaffold was soaked into Poly (ethylene glycol)-diacrylate (PEG)/LAP solution for 5 min. After PEG infiltration, the scaffold was photocrosslinked again to form the hybrid scaffold (GH+PEG). (**B**). Compressive modulus of GH and GH+PEG scaffolds. (*n* = 4 scaffolds). ** *p* < 0.01. (**C**). The appearance of different hydrogel scaffolds before (top panel) and after (bottom panel) the treatment with papain.

As shown in Figure 1A, GH hydrogels were first formed through photo-crosslinking, then soaked in uncured PEGDA/LAP solution for 5 min, allowing for infiltration. After that, the construct was subjected to illumination to cure infiltrated PEGDA. The impregnation of PEG significantly increased the mechanical strength of GH scaffolds (Figure 1B). To test whether the PEG scaffold was formed within the GH, papain solution, which digests gelatin, was used to treat different constructs. As shown in Figure 1C, after 24 h of treatment, GH scaffolds were not able to maintain the original structure and became thinner and softer. PEG scaffolds did not respond to papain treatment as expected. Interestingly, the PEG-infiltrated GH (GH+PEG) scaffolds could preserve the original dimension, suggesting PEG distributed thoroughly within GH.

### 2.2. Impregnation of PEG Affects the Distribution of GAGs in GH Scaffolds

**Figure 2:**
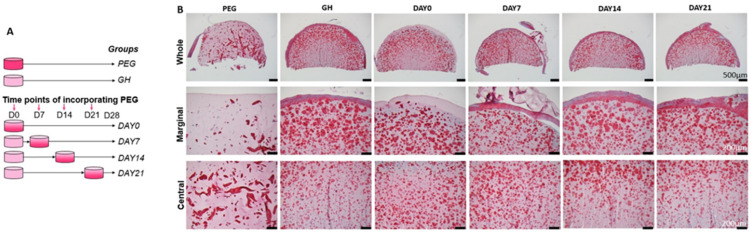
(**A**). Schematic showing the group information. At different points during 28 days of chondrogensis, PEG was incorporated into GH scaffold. (**B**). Safranin O staining to assess GAG deposition in different groups. DAY0, 7, 14, 21 indicated that PEG was introduced at Day 0, 7, 14, 21 of chondrogenic culture. Bar = 500 μm in the top panel and 200 μm in the middle and bottom panels.

hBMSCs were seeded in PEG or GH scaffolds and then subjected to chondrogenic culture for 4 weeks. Four other groups were also included, in which PEG was infiltrated into GH at different time points (Figure 2A). DAY 0, 7, 14, 21 indicated that PEG was introduced at Day 0, 7, 14, 21 of chondrogenic culture. After 28 days, safranin O was used to examine the GAGs deposition. Compared to GH, PEG showed a poor capacity to promote GAGs production (Figure 2B). In the samples from the GH group, we noticed that there were more GAGs in the margin area than in the central area. Interestingly, when PEG was infiltrated into GH on days 0 and 7, it promoted a uniform GAGs deposition, which was not seen in DAY14 and DAY21 groups (Figure 2B and Figure S1). The results from IHC for collagen type II (COL2) further confirmed that impregnating PEG at day 0 resulted in more deposition of COL2 in the central area than that performed at day 21 (Figure S2).

**Figure 3:**
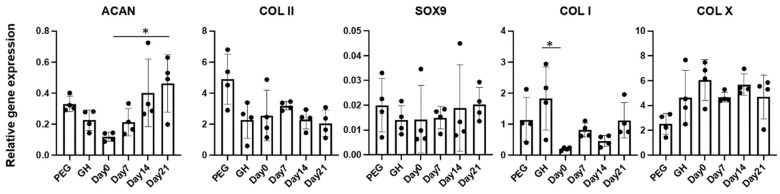
Relative expression levels of selected genes. Data was normalized to the housekeeping gene GAPDH (set as 1) (n = 4 cartilage tissues). * *p* < 0.05.

We next assessed the gene expression levels in different groups (Figure 3). Expression levels of *SOX9, COLII* and *COLX* were similar in all groups. However, we noticed that the earlier introduction of PEG resulted in lower expression levels of *ACAN*. Interestingly, cartilage tissues from Day0 group also displayed the lowest *COL1* in all groups.

Taken together, we introduced PEG into GH at day 0 in all experiments below.

### 2.3. Higher PEG Dose Allows More GAG Production in the Central Area of the Scaffold

**Figure 4:**
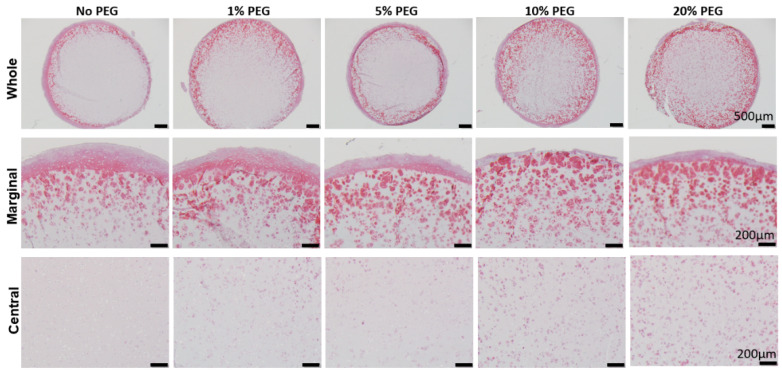
Safranin O staining to assess GAG deposition in different groups. 1–20% (*w*/*v*) indicated the concentration of uncured PEG solution that GH was dipped into. Bar = 500 μm in the top panel and 200 μm in the middle and bottom panels.

We next tested the influence of PEG concentration on the efficacy of promoting GAG deposition in the central area. As shown in Figure 4, soaking GH scaffolds in 20% PEGDA for 5 min resulted in a chondrogenesis with most uniform GAGs distribution in all tested groups.

### 2.4. PEG Infiltration Reduces Cell Proliferation Potential

**Figure 5:**
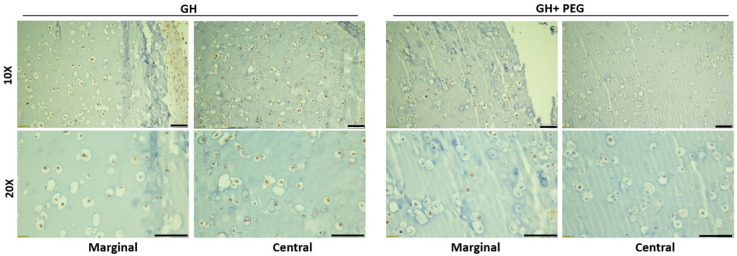
ki67 immunohistochemistry (IHC). Bar = 100 μm.

Next, we examined if the difference in cell proliferation accounted for the variable GAG distribution in different scaffolds. As shown in Figure 5 and Figure S3, cells in the margin and central areas of GH samples displayed high and similar proliferation potential, indicated by ki67 IHC. In the GH+PEG group, there were fewer ki67 positive cells in the center area than the margin area.

### 2.5. hBMSCs Migration Is Not Observed

**Figure 6:**
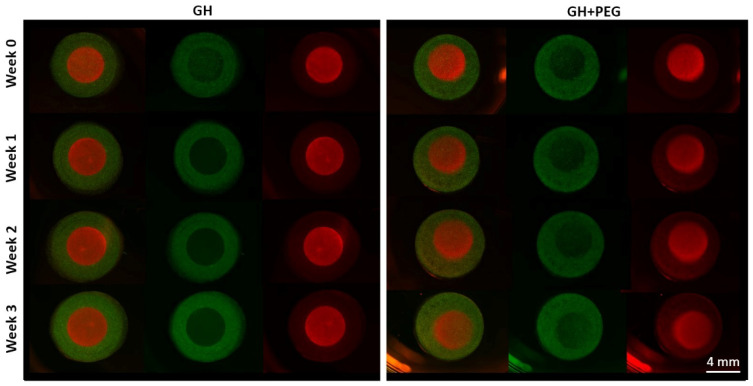
Cell migration test. Cells in the ring and disc insert were labeled with DiO (green) and DiI (red) separately.

To understand why there was more GAG in the central area after PEG infiltration, we first examined whether more cells migrated from the central area to the margin area. However, in the cell migration model that we created here, we did not observe cells crossing from the disc to the ring, or vice versa (Figure 6).

### 2.6. PEG-Infiltrated GH Hydrogel Integrates with Host Cartilage

**Figure 7:**
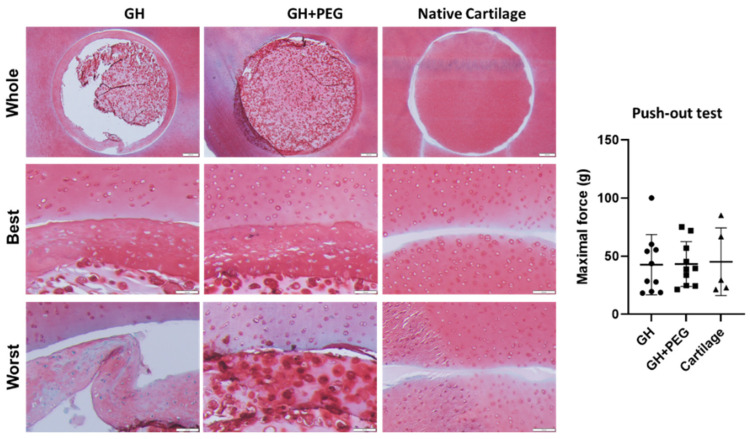
In vitro repair experiment with bovine cartilage explant. H&E staining was used to assess tissue integration. The maximal force during the push-out test was recorded and compared among the three groups. Circles, squares, and triangles represent individual data points in GH, GH+PEG, and cartilage groups, respectively.

To assess the reparative capacity, cartilage tissues created from hBMSCs within GH or GH+PEG scaffolds were implanted into bovine cartilage explants. As shown in Figure 7, the margin portion of cartilage tissue from GH group attached well with native cartilage. However, the inner portion detached during histological processing, implying different properties between the margin and central areas. In contrast, cartilage constructs from the GH+PEG group maintained the original structure and filled the defect. In the push-out test, we did not observe a difference in the maximal force during the test among the three groups.

## 3. Discussion and Conclusions

This study was conducted to develop scaffolds that are chondrosupportive and possess mechanical strength to support hBMSC chondrogenesis for articular cartilage tissue. The incorporation of PEG into pre-formed gelatin-hyaluronic acid-based gels, at the beginning of chondrogenesis, resulted in the most uniform distribution of proteoglycan deposition in all tested groups. Moreover, combined gels, GH+PEG, demonstrated a non-inferior tissue integration compared with pure GH scaffold and native cartilage.

For cartilage tissue engineering, the scaffolds should promote cell differentiation and tissue formation and also preserve biocompatible, biodegradable, and nontoxic properties. There are two types of scaffolds based on their origins, namely natural and synthetic materials. The natural scaffolds, such as hyaluronic acid, collagen, gelatin, or fibrin, are structurally reciprocal to native cartilage ECM, thus typically giving better support for chondrogenesis [11]. On the other hand, synthetic materials, such as PEG, are superior in terms of mechanical properties yet provide inadequate biological support [12]. Therefore, combining natural and synthetic biomaterials represents an often-used strategy to achieve desired properties to support cartilage regeneration. Specifically, our previous study showed that GH displayed a superior capacity to promote the chondrogenesis of hBMSCs, which however were also much softer than native cartilage. Therefore, our original idea was to initiate hBMSC chondrogenesis within GH scaffolds before introducing PEG to reinforce them. By this way, we can overcome the insufficiency of GH in mechanical strength and the limitations of PEG in supporting chondrogenesis. A technical challenge was how to include PEG polymers into hBMSCs-loaded GH constructs in the middle of chondrogenesis. We adapted a previous method developed in our lab [10], in which monomers and photoinitiators were first penetrated into native cartilage and then in situ cured within the tissue. A similar strategy was also reported by other research groups [13,14]. The advantage of this method is we can incorporate another type of biomaterials into pre-made scaffolds at any time. We previously found that five minutes of dipping was adequate for PEGDA penetration in a chip of 5 mm diameter and 2 mm height [10], and herein we demonstrated the stable form of PEG-infiltrated gels with greater mechanical stiffness constructs. In addition, chondrocyte culture in stiffer matrix material was found to be positively impact to chondrogenesis regarding the nature of articular cartilage as a weight-bearing structure [15].

In this study, our original plan was to allow the initiation of hBMSC chondrogenesis within GH scaffolds, before PEG was introduced. Therefore, our first study was to determine the best timing to infiltrate PEGDA. The first interesting finding was that after 28 days of chondrogenic culture, more GAGs were seen in the margin area of hBMSCs-loaded GH scaffolds than in the center. In our previous study, we did not notice such a phenomenon. A possible reason is the location of the slides that were used. In this study, we specifically focused on the area in the middle of the scaffolds, while in our previous study, the slides were from the surface area. Our results here were actually corresponding to Wu et al., who found that hollow cavity was usually present in biodegradable cartilage scaffolds larger than 2 mm [16], and this impeding effect was more obvious in denser, stiffer hydrogel network, as shown in Zhao and coworkers’ study [17]. To partially solve the issue, dynamic culture on an orbital shaker has used to increase the central deposition of GAGs [18].

A surprising finding was that uniform GAGs production was achieved when PEG was introduced at the beginning of chondrogenesis, which did not agree with our hypothesis that the early introduction of PEG would impair chondrogenesis. Then, we carefully analyzed tissue structures from different groups. Without using PEG, a very dense layer of newly formed cartilage tissues covered the entire constructs. Moreover, this extra structure was thicker when PEG was introduced later. Therefore, it was thought that PEG infiltration prevented the formation of this tissue. In addition, we also observed that early PEG incorporation resulted in more GAG deposition in the center area. Therefore, we assumed that the thick cartilage layer outside of the scaffolds prevented the nutrient from the culture medium to the center of the scaffolds. However, ki67 IHC results showed that cells within GH displayed higher proliferation potential than GH+PEG in both the marginal and central areas. Therefore, it seems like the dense outer layer did not affect the penetration of nutrients.

Since we also noticed fewer cells in the central area of GH than in GH+PEG, we next examined if cell migration is one mechanism resulting in the difference in GAG deposition. Previously, it had been reported that MSCs could migrate in hyaluronic acid [19] and gelatin [20]-based scaffolds. However, in the method that we used in this study to assess cell migration, we did not see MSCs that crossed from ring to disc or vice versa. The reason that we made the ring and disc separately was that we needed to label cells with different colors to track their potential movement. Therefore, there was a physical barrier between the two scaffolds, which may potentially limit cell migration. In the future, live-cell imaging can be included to monitor the cell movement in the scaffolds [21], which will answer whether cell migration out of the central area is the reason resulting in less GAG production.

Lastly, we investigated tissue integration using fresh cartilage discs from cow knees. Failure to integrate with native cartilage would eventually result in the degradation of the implanted cartilage tissues or surrounding tissue [22]. In this study, we noticed that the dense out layer in the GH group integrated strongly with native cartilage tissues, even going through the process of histology. This result was consistent with previous studies that ECM-derived components optimize the cellular microenvironment, which results in tissue integration [23]. However, the structure detached from the original GH scaffold, indicating the potential issue of GH in repairing chondral injury. Interestingly, we saw the samples from the GH+PEG group maintained their integration with native cartilage as an intact structure, suggesting its superiority to GH. However, in the push-out test, we did not observe a significant difference in maximal force among the three tested groups. To further enhance integration between native cartilage and implants, we may introduce additional treatments, such as lysyl oxidase [24].

There are several limitations in this study. First, we used two scaffolds instead of a uniform scaffold to test cell migration, which potentially created a barrier to block cell migration. Second, in the in vitro repairing study, a high variation within groups was observed, which was partially due to the use of cartilage explants harvested from different locations of the knee joint. Lastly, the mechanism of more uniform matrix distribution within GH+PEG scaffolds has not been fully elucidated.

In conclusion, in this study, we attempted to create simple hybrid scaffolds that support chondrogenesis with better mechanical properties by infiltrating synthetic properties of PEG into gelatin and hyaluronic acid-based scaffolds. A uniform matrix distribution was observed throughout GH+PEG scaffolds, which also supported in vitro cartilage repair and integration. Further study will test its chondral repairing capacity in animals.

## 4. Materials and Methods

### 4.1. Isolation of hBMSCs

With Institutional Review Board approval (University of Pittsburgh), hBMSCs were isolated from the bone marrow of femoral heads received from donors who underwent total hip arthroplasty as surgical waste. Growth medium (GM, α-MEM containing 10% fetal bovine serum (FBS, Invitrogen, Carlsbad, CA, USA), 1% Antibiotic-Antimycotic (Invitrogen), and 1 ng/mL FGF-2 (RayBiotech, Norcross, GA, USA)) was used to resuspend freshly isolated cells.

Cells were plated into 150 cm^2^ tissue culture flasks at Passage 0, with media changes every 3 to 4 days, then passaged once reaching 80% confluence. Stemness of hBMSCs was determined by their osteogenic, adipogenic, and chondrogenic differentiation capabilities, which was proven by different staining methods, including Alizarin red, safranin O, and oil red O, respectively. hBMSCs pooled from 3 patients (54 years old female, 52 years old female and 57 years old male) were used in this study. All experiments were performed with passage 4-5 (P4-5) hBMSCs.

### 4.2. Preparation of Scaffold Materials

The photoinitiator lithium phenyl-2,4,6-trimethylbenzoylphosphinate (LAP) was synthesized according to the protocol developed by Fairbanks et al. [25]. PEGDA was purchased (AdvancedBiomatrix, Carlsbad, CA, USA). PBS (Invitrogen) was used to make PEGDA solution with a variety of concentrations, namely 1%, 5%, 10%, and 20%. 0.15% (*w*/*v*) LAP was added as the photoinitiator. Methacrylate gelatin (GelMA) was synthesized according to a procedure previously developed in our lab [8]. In brief, Gelatin (Sigma-Aldrich, St. Louis, MO, USA) was fully dissolved in deionized H_2_O in a shaker at 37 °C, and methacrylic anhydride was then added. The mixture was placed in a 37 °C shaker at 150 rpm for 24 h and dialyzed for 4 days against H_2_O at room temperature using 2000 NMWCO dialysis tubing (Sigma-Aldrich). After lyophilization, the GelMA product was stored in a desiccator for future use. Photocrosslinkable Hyaluronic acid (HA, 100–150 kDa) powder was purchased (AdvancedBiomatrix). Hybrid hydrogel (GelMA/HA or GH) was created by dissolving in Hank’s Balanced Salt Solution (HBSS) at (GelMA:HA, *w*/*v*) 9%:1%. The photoinitiator, LAP, was then added (0.15% *w*/*v*) and mixed until fully dissolved [9].

### 4.3. Mechanical Testing

As shown in Figure 1A, GH hydrogels were first formed through photo-crosslinking, then soaked in uncured PEGDA/LAP solution for 5 min, allowing for infiltration. After that, the construct was subjected to illumination to cure infiltrated PEGDA, forming GH+PEG scaffolds. To assess the mechnical property of GH after PEG impreganation, an electromechanical tester with 1000 g load cell (ElectroForce 3200, Bose, Eden Prairie, MN, USA) was used. Specifically, cylinderical GH or GH+PEG scaffolds with 2 mm height and 5 mm diameter were placed between stainless steel discs and then subjected to 10% unconfined compression using a constant rate (0.01 mm/s). Compressive moduli of these scaffolds were calculated based on the slope of force versus displacement plots.

### 4.4. Encapsulation of hBMSCs into Scaffolds for Chondrogenesis

Two types of scaffolds, PEG and GelMa/HA, were prepared as described above. P4-5 hBMSCs pellets were resuspended in the uncured solution with a final density of 20 × 10^6^ cells/mL. The suspension was then added into cylindrical void molds with 2 mm heights. They were then cured with visible light (Mega Light CL, DBI America, Lutz, FL, USA), producing light at 430 nm–490 nm with a total exposure time of 2 min. Scaffold modification was done by soaking GelMA/HA gels into PEG solution for 5 min which is enough for PEG to penetrate the entire construct. Then, they were subjected to illumination for one and half minutes under visible light to cure them (GH+PEG scaffolds). All of the constructs were cultured in chondrogenic medium consisting of DMEM with high glucose, 1% penicillin-streptomycin, 0.1 mM dexamethasone, 50 mg/mL ascorbate-2-phosphate, and 40 mg/mL L-proline (Sigma-Aldrich), 1×insulin-transferrin-selenium (Invitrogen), and 10 ng/mL transforming growth factor beta-3 (TGFβ3) for 4 weeks before further analysis or explant culture.

### 4.5. Explant Isolation and Culture

Articular cartilage explants were harvested from the knee joint of a newborn bovine (from a local slaughterhouse) within 24 h after being sacrificed. The cartilage disks of 6 mm diameter and 3–4 mm thickness was punctured with 4 mm of inner diameter to create cartilage ring. Different cartilage-like tissues in vitro generated from hBMSCs-encapsulated GH or GH+PEG were implanted into bovine cartilage explant ring and cultured for the next two weeks in chondrogenic-maintaining medium (DMEM with high glucose, 1% penicillin-streptomycin, 0.1 mM dexamethasone, 50 mg/mL ascorbate-2-phosphate, and 40 mg/mL L-proline (Sigma-Aldrich), 1×insulin-transferrin-selenium (Invitrogen), and 0.5 ng/mL TGF-β3). Two explants were collected for further histological evaluation. Four explants were then used for push-out testing, and implants were used for later PCR experiments.

### 4.6. Analysis of Gene Expression by Real-Time Reverse Transcription PCR (RT-PCR)

To isolate RNA, constructs were crushed in the Qiazol reagents (Invitrogen) in a 1.5 mL Eppendorf tube using a plastic pestle. RNA was purified using RNeasy Plus Mini Kit (Qiagen, Germantown, MD, USA). Reverse transcription was done using SuperScript^®^ VILO™ cDNA Synthesis Kit (Invitrogen) according to the manufacturer’s protocol. SYBR Green Reaction Mix (Applied Biosystems, Foster City, CA, USA) with a StepOne-Plus thermocycler (Applied Biosystems) was used to run the real-time PCR, and gene expression levels of Sox 9, collagen types I (*COLI*), II (*COLII*) and X (*COLX*), aggrecan (*ACAN*), and matrix metalloproteinase 13 (*MMP13*) were analyzed. Of note, ribosomal protein L13a (RPL13A) was used to normalize all values using the 2^−ΔΔCt^ method.

### 4.7. Histology and Staining

Different hBMSCs-derived cartilage tissues were fixed in 10% neutral buffered formalin (Fisher Scientific, Pittsburgh, PA, USA) for 1 day, then underwent alcohol dehydration, were then paraffin-embedded, and 6 μm sections were sectioned. Staining with Safranin O/Fast Green was done to evaluate proteoglycan deposition.

### 4.8. Immunohistochemistry (IHC)

Vectastain ABC kit and the NovaRED peroxidase substrate kit (Vector Labs, Burlingame, CA, USA) were used for IHC. Briefly, after deparaffinization and rehydration, sections were subjected to antigen retrieval. Antigen was retrieved by heat-mediated antigen retrieval (eBioscience, San Diego, CA, USA) in 90 °C for 20 min. Endogenous peroxidase was inactivated with 3% (*v*/*v*) hydrogen peroxide in methanol for 10 min at room temperature. After being blocked with 1% horse serum in PBS for 45 min, the slides were incubated with primary antibody against ki67 (ThermoFisher, Waltham, MA, USA) or collagen type II (Abcam, Boston, MA, USA) at 4 °C overnight. Following this, slides were washed in PBS and incubated in biotinylated secondary antibody for 30 min, washed three times, and then incubated in Vectastain Elite ABC reagent for 30 min. Finally, peroxidase substrate was added and incubated for an appropriate time, dependent upon the different targets, for visualization. After staining, slides were dehydrated and mounted with glass coverslips.

### 4.9. Push-Out Test

Integration of cartilage implants into native cartilage explants was evaluated using a mechanical tester (Bose Electroforce model 3230 Series II) to conduct the push-out test. The samples were placed on customized central hole plate with diameter of 5 mm, as the void space was large enough so that the implant could move downward while pushing. A metal plunger (2 mm diameter) connected to the mechanical tester was used to push the implant. The maximum force that was able to make the implant fall down was recorded. The displacement rate was 0.1 mm/s.

### 4.10. Statistical Analysis

All data was measured as mean ± standard deviation and statistical analysis was performed using two-way independent analysis of variance (ANOVA). A threshold of *p* < 0.05 was used to determine statistical significance.

## Data Availability

Not applicable.

## Supplementary Materials

The following supporting information can be downloaded at: https://www.mdpi.com/article/10.3390/gels8120794/s1, Figure S1. Assessing GAG distribution with semi-quantitative scores; Figure S2: Collagen type II immunohistochemistry (IHC); Figure S3: Qualifying ki67 positive cells in each field.
