# Supplementary material for: PEG Reinforced Scaffold Promotes Uniform Distribution of Human MSC-Created Cartilage Matrix
Source: Gels. 2022 Dec 3;8(12):794. doi: 10.3390/gels8120794 (PMC9778361; doi:10.3390/gels8120794)
Supplement: Supplementary file 1 [file gels-08-00794-s001.zip › gels-1998518-supplementary.pdf]

PEG Reinforced Scaffold Promotes Uniform Distribution of Human MSC-created Cartilage Matrix

Kanyakorn Riewruja [1,2], Alyssa M Aguglia [1,3], Sophie Hines [1], Meagan J. Makarcyzk [1,3], Sittisak Honsawek[2], and Hang Lin [1, 3]*

[1] Department of Orthopaedic Surgery, University of Pittsburgh School of Medicine; 450 Technology Drive, Rm 217, Pittsburgh, Pennsylvania, 15219, USA

[2] Osteoarthritis and Musculoskeleton Research Unit, Faculty of Medicine, Chulalongkorn University, King Chulalongkorn Memorial Hospital, Thai Red Cross Society, Bangkok 10330, Thailand

[3] Department of Bioengineering, University of Pittsburgh Swanson School of Engineering; 450 Technology Drive, Rm 217, Pittsburgh, Pennsylvania, 15219, USA

* Correspondence: hal46@pitt.edu;

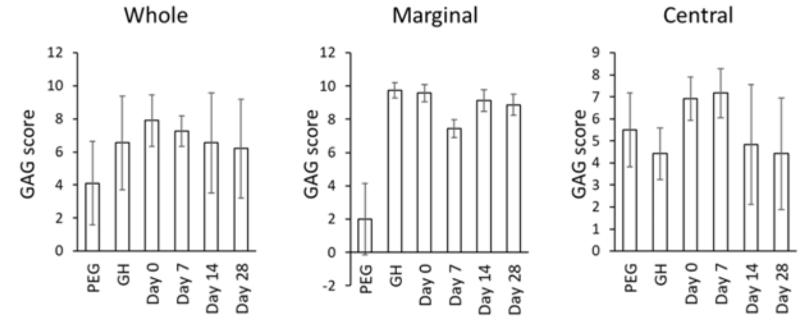

Figure S1. Assessing GAG distribution with semi-quantitative scores. Based on the staining intensity of the whole, marginal and central areas in each image of Figure 2, a score from 0 (no GAG) to 10 (most GAG) was given (n=8 fields).

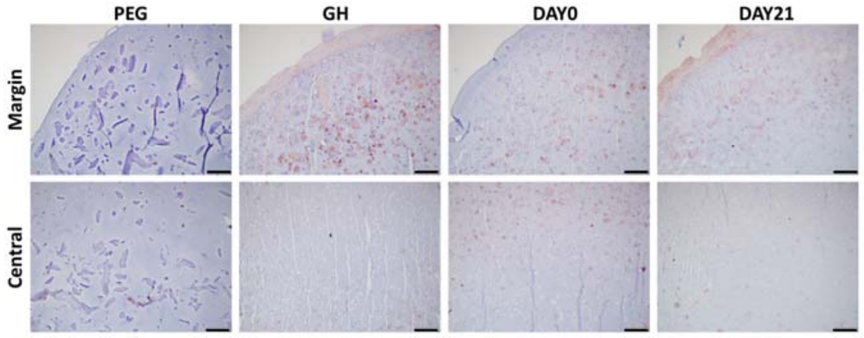

Figure S2: Collagen type II immunohistochemistry (IHC). Bar=100μm.

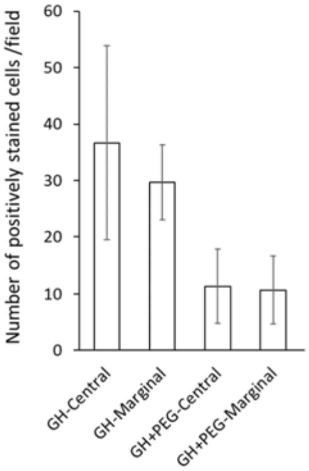

Figure S3: Qualifying ki67 positive cells in each field (512µmx320µm). N=3.
